# Integrating the Neutrophil-to-Lymphocyte Ratio into a Clinicopathological Nomogram for Event-Free Survival Prediction in Cisplatin-Treated Muscle-Invasive Bladder Cancer

**DOI:** 10.3390/cancers18132054

**Published:** 2026-06-24

**Authors:** Mariona Figols, Andrea González, Maria Fernandez-Saorín, Ana Bautista, Olatz Etxaniz, Ester Ruz, Jose Luis Gago, Daniela Gómez-Díaz, Juan Carlos Pardo, Marta Galí, Sergi Bernal, Cristina Camps, Lorena Rifa, Montserrat Domenech, Vicenç Ruiz de Porras, Anna Esteve, Albert Font

**Affiliations:** 1 Medical Oncology Department, Althaia Xarxa Assistencial Universitària de Manresa, C/Dr. Joan Soler 1–3, 08243 Manresa, Spain; ambautista@althaia.cat (A.B.); dgomezd@althaia.cat (D.G.-D.); mgali@althaia.cat (M.G.); lrifa@althaia.cat (L.R.); mdomenech@althaia.cat (M.D.); 2 PhD Programme in Medicine and Biomedical Sciences, Doctoral School, University of Vic-Central University of Catalonia (UVic-UCC), C/Dr. Junyent 1, 08500 Vic, Spain; 3 Faculty of Medicine, University of Vic-Central University of Catalonia (UVicUCC), Can Baumann Ctra de Roda 70, 08500 Vic, Spain; 4 Medical Oncology Department, Catalan Institute of Oncology, Camí de les Escoles, s/n, 08916 Badalona, Spain; andreagonzalez@iconcologia.net (A.G.); oetxaniz@iconcologia.net (O.E.); jcpardor@iconcologia.net (J.C.P.); ccguevara@iconcologia.net (C.C.); aesteve@iconcologia.net (A.E.); afont@iconcologia.net (A.F.); 5 CARE Program, Germans Trias i Pujol Research Institute (IGTP), Camí de les Escoles, s/n, 08916 Badalona, Spain; mfernandezs@igtp.cat; 6 Badalona Applied Research Group in Oncology (B·ARGO), Catalan Institute of Oncology, Camí de les Escoles, s/n, 08916 Badalona, Spain; 7 Urology Department, Althaia Xarxa Assistencial Universitària de Manresa, C/Dr. Joan Soler 1–3, 08243 Manresa, Spain; eruz@althaia.cat; 8 Urology Department, University Hospital Germans Trias i Pujol, Camí de les Escoles, s/n, 08916 Badalona, Spain; jlgago.germanstrias@gencat.cat (J.L.G.); sbernals.germanstrias@gencat.cat (S.B.); 9 GRET and Toxicology Unit, Department of Pharmacology, Toxicology and Therapeutic Chemistry, Faculty of Pharmacy and Food Sciences, University of Barcelona, 08028 Barcelona, Spain

**Keywords:** muscle-invasive bladder cancer, neoadjuvant therapy, prognosis, nomogram, risk assessment, survival analysis

## Abstract

Background/Objectives: Neoadjuvant cisplatin-based chemotherapy (NAC) followed by radical cystectomy (RC) is a standard treatment for cisplatin-eligible patients with muscle-invasive bladder cancer (MIBC), yet baseline tools to refine prognostic stratification remain limited. We aimed to develop and internally validate a clinicopathological nomogram integrating the neutrophil-to-lymphocyte ratio (NLR) to estimate event-free survival (EFS) in patients with MIBC treated with NAC. Methods: We retrospectively analyzed 210 patients with cT2–T4aN0–1M0 MIBC treated with cisplatin-based NAC at two Spanish institutions between 2010 and 2021. Candidate predictors included demographic, clinicopathological, and routine laboratory variables. A multivariable Cox model with backward selection based on the Akaike information criterion (AIC) was used to derive the final model, and internal validation was performed using 1000 bootstrap resamples. Results: Sex, age, prior non–muscle-invasive bladder cancer (NMIBC), and NLR were retained in the final nomogram. The model showed moderate discrimination, with a Harrell’s c-index of 0.60 and an optimism-corrected c-index of 0.58. The nomogram stratified patients into low-, intermediate-, and high-risk groups, with median EFS not reached, 47.5 months, and 18.0 months, respectively. High-risk patients also showed lower pathological complete response (pCR) rates. Conclusions: This exploratory nomogram integrates an accessible systemic inflammatory marker with baseline clinical variables to identify patients with poorer outcomes despite NAC. External validation in contemporary cohorts is warranted before clinical implementation.

## Simple Summary

Patients with muscle-invasive bladder cancer who are fit for cisplatin usually receive chemotherapy before bladder-removal surgery. However, not all patients benefit from this approach, and some still relapse or die after treatment. In this study, we investigated whether information available before chemotherapy could help identify patients with a higher risk of poor outcomes. We analyzed 210 patients treated at two Spanish hospitals and developed a risk model based on age, sex, previous non-muscle-invasive bladder cancer, and a simple blood-test measure called the neutrophil-to-lymphocyte ratio, which reflects the balance between inflammation and immune cells. This model classified patients into low-, intermediate-, and high-risk groups. Patients in the high-risk group had a shorter time without relapse or death and were less likely to have no residual cancer in the bladder after treatment. The model is exploratory and should not yet be used to guide treatment decisions. However, after validation in other patient groups, it may help improve risk assessment and support more personalized treatment strategies.

## 1. Introduction

Neoadjuvant cisplatin-based chemotherapy (NAC) followed by radical cystectomy (RC) remains a standard treatment for cisplatin-eligible patients with muscle-invasive bladder cancer (MIBC) [1,2]. More recently, the addition of perioperative immunotherapy to cisplatin-based NAC has demonstrated improved clinical outcomes, further reshaping the therapeutic landscape of localized MIBC [3]. Despite this multimodal approach, nearly half of patients experience recurrence, and the 5–8% overall survival (OS) benefit of NAC is limited to those achieving a pathological complete response (pCR) [4,5,6,7]. Patients who do not obtain pCR are exposed to treatment toxicity and surgical delays, both associated with inferior OS [8,9,10]. Therefore, identifying patients most likely to benefit from NAC remains an unresolved challenge.

Numerous clinicopathological, molecular, and blood-based biomarkers have been investigated to predict response and survival outcomes in patients with MIBC treated with NAC [11,12,13]. However, no single biomarker has been consistently incorporated into routine clinical practice. In this context, multivariable risk models and nomograms may offer a practical strategy to integrate complementary prognostic information and support individualized treatment decision-making [14]. For example, a recently developed nomogram incorporating tumor origin, histology, clinical stage, and tumor size predicted pCR in NAC-treated MIBC patients with promising performance, highlighting the potential utility of clinically interpretable tools in this setting [15].

Systemic inflammation has also emerged as a relevant biological and prognostic feature in bladder cancer. Among routinely available inflammatory biomarkers, the neutrophil-to-lymphocyte ratio (NLR) is one of the most widely studied. Elevated pretreatment NLR has been associated with lower pCR rates, shorter survival, and poorer outcomes after RC in patients receiving NAC [16]. Similarly, preoperative NLR has been correlated with adverse pathological features, including extravesical extension and nodal involvement, as well as recurrence and mortality [17]. A recent meta-analysis including 8448 patients with MIBC undergoing RC confirmed the adverse prognostic impact of elevated NLR on survival outcomes [18]. Other systemic inflammatory indices, such as the platelet-to-lymphocyte ratio, systemic immune-inflammation index, and derived neutrophil-to-lymphocyte ratio, have also been associated with worse outcomes after RC, including in NAC-treated cohorts [19]. However, NLR was selected for the present model because it is among the most widely investigated inflammatory biomarkers in patients receiving NAC followed by RC, and it is simple, routinely available, and clinically interpretable. In addition, models combining inflammatory biomarkers with clinicopathological features have improved the prediction of non–organ-confined disease at RC [20].

Our group previously developed a nomogram and risk score from a retrospective cohort of 247 NAC-treated MIBC patients to predict bladder cancer-specific survival (BCSS). The model incorporated four variables: variant histology, prior non–muscle-invasive bladder cancer (NMIBC), female sex, and hydronephrosis, and stratified patients into low-, intermediate-, and high-risk groups with 5-year BCSS rates of 72%, 53%, and 15%, respectively. Notably, high-risk patients had a pCR rate of only 8% versus 38% in the low-risk group, underscoring the need to consider alternative therapies for those unlikely to benefit from NAC [21]. These findings support the biological and clinical relevance of integrating systemic inflammatory biomarkers into prognostic models for MIBC.

Building on this previous work, the present study aimed to develop and internally validate a prognostic nomogram to estimate 2- and 5-year event-free survival (EFS) in patients with MIBC treated with cisplatin-based NAC followed by RC. By integrating the NLR, an accessible systemic inflammatory biomarker, with baseline clinicopathological variables, we sought to improve baseline prognostic stratification and provide a framework for future risk-adapted treatment strategies in MIBC.

## 2. Materials and Methods

### 2.1. Study Design and Patient Population

This retrospective multicenter cohort study included adult patients with cT2–T4aN0–1M0 MIBC treated with cisplatin-based NAC followed by RC at two Spanish hospitals between January 2010 and December 2021. Follow-up was updated until July 2024. Patients were identified from institutional registries and electronic medical records. Clinical management was performed according to national and international guidelines in place at the time of treatment.

### 2.2. Eligibility Criteria

Inclusion criteria were: histologically confirmed urothelial carcinoma of the bladder with a predominantly urothelial component on TUR of bladder tumor; clinical stage cT2–T4aN0–1M0 based on thoracoabdominopelvic computed tomography; cisplatin eligibility according to Galsky criteria [22], receipt of more than one cycle of cisplatin-based NAC; and availability of baseline clinicopathological and laboratory data with complete follow-up information.

Exclusion criteria were: predominant non-urothelial histology, metastatic disease at diagnosis, treatment with non-cisplatin-based neoadjuvant regimens, missing relevant clinical or laboratory data, or unavailable follow-up information.

### 2.3. Data Collection and Variables

Treatment consisted of 3–4 cycles of cisplatin-based NAC followed by RC, according to institutional protocols. Pathological staging (pTNM) was obtained from RC specimens and classified according to the AJCC TNM classification used at the time of treatment.

Baseline variables collected from medical records included age, sex, cTNM (T2N0 vs. ≥T3 and/or N1), histology on TUR (urothelial vs. variant), presence of lymphovascular invasion (LVI), prior NMIBC and hydronephrosis on pre-NAC imaging.

Baseline laboratory parameters included hemoglobin, platelet count, and neutrophil-to-lymphocyte ratio (NLR). Laboratory values were obtained within 30 days before NAC initiation. Hemoglobin was categorized as <12 versus ≥12 g/dL, platelet count as <300 versus ≥300 × 10^9^/L, and NLR was calculated as the absolute neutrophil count divided by the absolute lymphocyte count. NLR was evaluated both as a continuous variable and as a dichotomous variable using the cohort median cutoff of 2.78, given the lack of a universally accepted threshold in patients with MIBC treated with NAC followed by RC. This median-based cutoff was selected to provide a balanced distribution of patients between groups and to avoid outcome-driven cutoff optimization, which could increase the risk of overfitting in a retrospective cohort. The time from TUR to NAC initiation (TTNAC) was recorded in weeks and categorized as <6 versus ≥6 weeks.

OS was defined as the time in months from TUR to death from any cause or last follow-up. EFS was defined as the time in months from TUR to any of the following: disease progression precluding RC, recurrence after RC, omission of RC for non-oncological reasons, death from any cause, or last follow-up. Follow-up was administratively censored at 5 years after RC. Additional outcomes included pTNM, date of recurrence, bladder cancer–specific death, and vital status.

### 2.4. Statistical Analysis

OS and EFS were estimated using the Kaplan–Meier method and compared using the log-rank test. Median survival times and 95% confidence intervals (CIs) were reported. Categorical variables were summarized as frequencies and percentages, and continuous variables as median and interquartile range (IQR), unless otherwise specified.

Candidate baseline predictors for EFS included age, sex, histology, LVI, prior NMIBC, hydronephrosis, clinical TNM stage, baseline hemoglobin, baseline platelet count, NLR, and TTNAC. Only variables available before or at NAC initiation were considered for development of the prognostic model.

A multivariable Cox proportional hazards regression model with backward stepwise selection based on the Akaike information criterion (AIC) was used to derive the final prognostic model. Hazard ratios (HRs) with 95% CIs were calculated. The proportional hazards assumption was assessed using Schoenfeld residuals. No statistically significant violation of the proportional hazards assumption was observed for any of the variables included in the final Cox model: sex (*p* = 0.172), age (*p* = 0.426), prior non-MIBC (*p* = 0.166), and NLR (*p* = 0.062). The global test was also non-significant (*p* = 0.119), supporting the adequacy of the proportional hazards assumption for the final model. These results are shown in Supplementary Figure S1. Discrimination of the final model was assessed using Harrell’s c-index. Time-dependent receiver operating characteristic (ROC) analyses were performed to estimate the area under the curve (AUC) for EFS prediction at 1, 2, 4, and 5 years. After the final model was obtained, a nomogram was constructed to estimate 2- and 5-year EFS probabilities. A risk score was then derived from the nomogram: each predictor was assigned points proportional to its relative contribution in the final Cox model (i.e., its regression coefficient scaled by the variable’s range); these points were summed into a total score that was then mapped onto the predicted 2- and 5-year EFS probabilities. Patients were stratified into low-, intermediate-, and high-risk groups according to score cut-offs defined post hoc from the observed event-free survival curves.

Internal validation of the final prognostic model was performed using bootstrap resampling with 1000 repetitions, and the optimism-corrected c-index was estimated. Calibration was assessed graphically by comparing predicted and observed EFS probabilities at 1, 2, 4, and 5 years. All statistical analyses were conducted using R software version 4.1.1. A two-sided *p*-value < 0.05 was considered statistically significant.

## 3. Results

### 3.1. Patients

**Figure 1:**
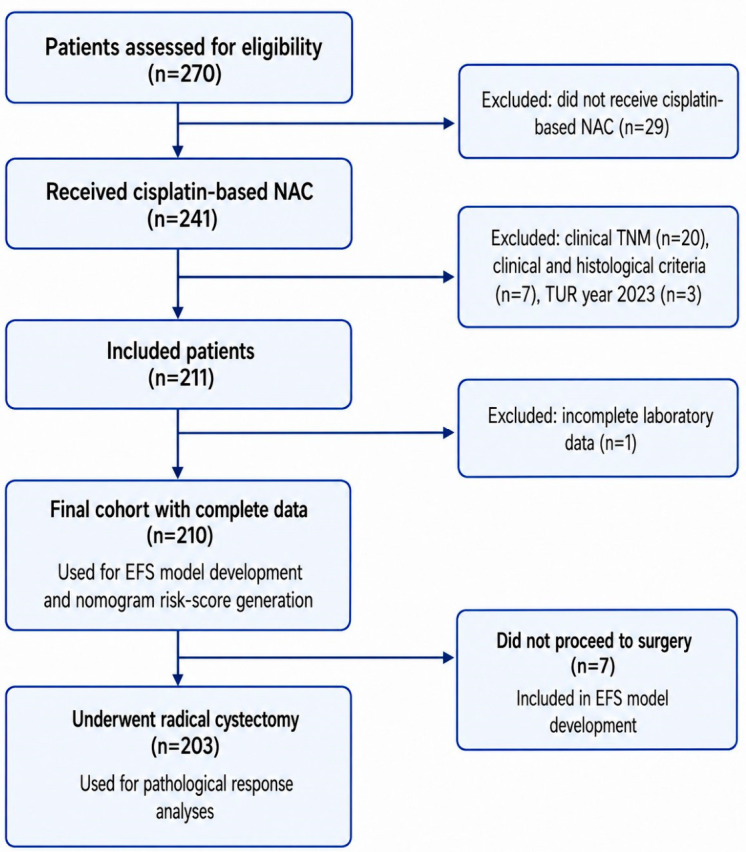
Patient selection flowchart. Flowchart showing the selection of patients with muscle-invasive bladder cancer (MIBC) treated with cisplatin-based neoadjuvant chemotherapy (NAC). The final analytical cohort included 210 patients and was used for event-free-survival (EFS) model development and nomogram risk-score generation. Among these patients, 203 underwent radical cystectomy and 7 did not proceed to surgery. Pathological response analyses were restricted to patients who underwent radical cystectomy (RC). TNM, tumor-node-metastasis; TUR, transurethral resection.

A total of 270 patients were assessed for eligibility between 2010 and 2021. After exclusions, 210 patients were included in the final analytical cohort (Figure 1). Most patients were male (n = 187, 89.0%), and the median age was 67 years (IQR, 62–74). Pure urothelial carcinoma was present in 176 patients (83.8%), whereas 34 patients (16.2%) had variant histology. Baseline clinical stage was cT2N0M0 in 64 patients (30.5%), cT3–4aN0M0 in 123 patients (58.6%), and cT2–4aN+M0 in 23 patients (11.0%). Most patients received cisplatin–gemcitabine (n = 195, 92.9%), while 15 patients (7.1%) received dose-dense MVAC (dd-MVAC). Baseline NLR was <2.78 in 104 patients (49.5%) and ≥2.78 in 106 patients (50.5%). Seven patients (3.3%) did not undergo RC. Among the 203 patients who underwent RC, complete resection was achieved in 190 patients (93.6%), whereas 13 patients (6.4%) had incomplete resection. Baseline characteristics, laboratory variables, treatment-related variables, and pathological outcomes are summarized in Table 1.

### 3.2. Outcomes

Of the 203 patients who underwent RC, 97 patients (47.8%) achieved pathological downstaging, defined as ypT0/Ta/Tis/T1, including 68 patients (33.5%) with pCR (ypT0N0). Pathological lymph node involvement was observed in 40 patients (19.7%). Baseline characteristics were generally balanced between responders (ypT0/Ta/Tis/T1) and non-responders (≥ypT2 and/or ypN+), as shown in Supplementary Table S1.

The median follow-up was 43 months (range, 20.2–76.7). At the time of analysis, 114 patients (54.3%) had died, including 80 patients (38.1%) from bladder cancer and 34 patients (16.2%) from non-cancer-related causes. Median OS was 58.7 months (95% CI, 47.5–NR), with estimated 2- and 5-year OS rates of 71% (95% CI, 65–77) and 50% (95% CI, 43–57), respectively. Median EFS was 48.5 months (95% CI, 37.7–NR), with estimated 2- and 5-year EFS rates of 62% (95% CI, 56–69) and 47% (95% CI, 41–55), respectively.

Pathological response, resection status, and NLR were significantly associated with both OS and EFS, whereas sex was significantly associated with EFS only (Supplementary Tables S2 and S3). Responders had a median OS that was not reached, compared with 26.0 months in non-responders, with corresponding 5-year OS rates of 70% and 34%, respectively (*p* < 0.001; Supplementary Figure S2A). Similarly, median EFS was not reached in responders, compared with 17.7 months in non-responders, with corresponding 5-year EFS rates of 69% and 31%, respectively (*p* < 0.001; Supplementary Figure S2B). Patients with baseline NLR < 2.78 had a median EFS that was not reached, whereas those with NLR ≥ 2.78 had a median EFS of 32.0 months (95% CI, 19.0–NR; *p* = 0.038).

### 3.3. Prognostic Nomogram for EFS

An initial multivariable Cox regression model included age, sex, lymphovascular invasion, prior NMIBC, hydronephrosis, clinical TNM stage, baseline hemoglobin, baseline platelet count, NLR, and TTNAC. After backward selection based on the Akaike information criterion, four variables were retained in the final prognostic model: sex, age, prior NMIBC, and NLR (Table 2). The final model showed moderate discrimination, with a Harrell’s c-index of 0.60 (95% CI, 0.54–0.66).

**Figure 2:**
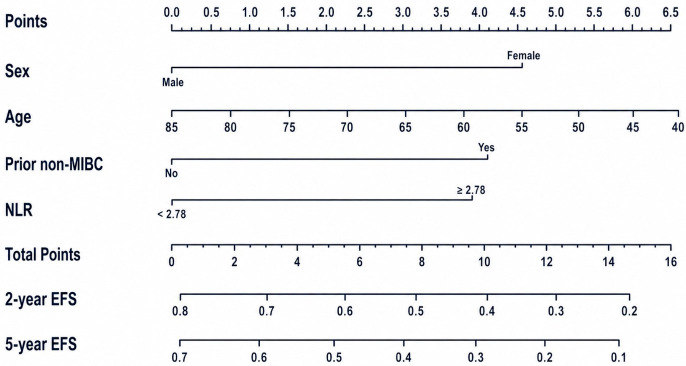
Nomogram for predicting 2- and 5-year event-free survival (EFS) in patients with MIBC treated with cisplatin-based NAC followed by radical cystectomy. The nomogram integrates four baseline variables: sex, age, prior non–muscle-invasive bladder cancer (NMIBC), and neutrophil-to-lymphocyte ratio (NLR).

A risk score was then developed based on the relative contribution of each variable in the final model (Figure 2). Younger age contributed the highest number of points, followed by female sex, prior NMIBC, and NLR ≥ 2.78. Patients were stratified into three risk groups: low risk (≤5 points; n = 81), intermediate risk (6–8 points; n = 83), and high risk (≥9 points; n = 46). As an exploratory secondary observation among patients who underwent surgery, the pCR rate decreased progressively across the low-, intermediate-, and high-risk groups: 42.5%, 31.2%, and 20.9%, respectively (*p* = 0.046).

**Figure 3:**
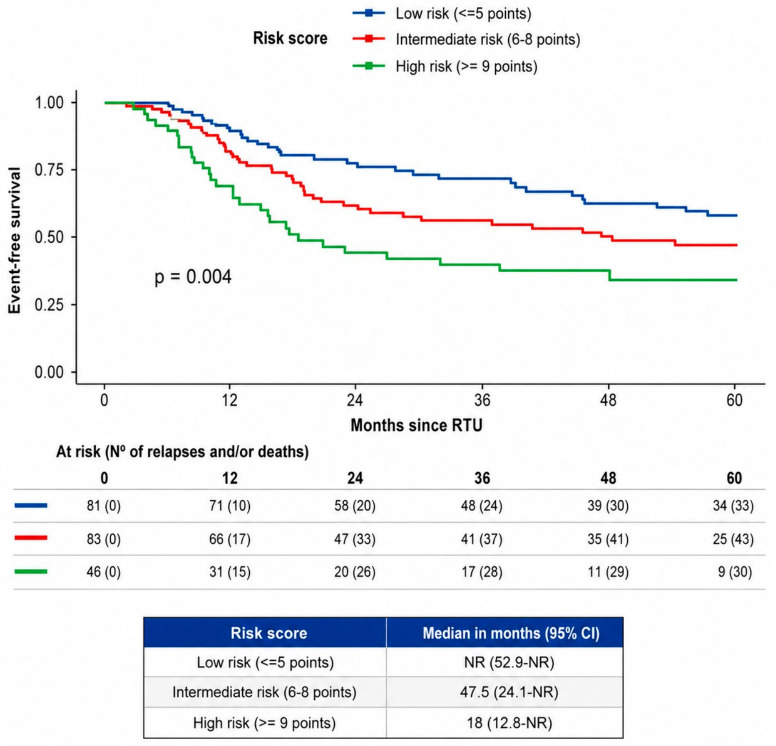
Kaplan–Meier estimates of event-free survival according to nomogram-derived risk groups. Patients with MIBC treated with cisplatin-based NAC followed by radical cystectomy were stratified into low-risk, intermediate-risk, and high-risk groups according to the prognostic risk score. The table below the plot shows the number of patients at risk and the cumulative number of relapses and/or deaths. EFS, event-free survival; RTU, transurethral resection.

The nomogram was used to estimate 2- and 5-year EFS probabilities (Figure 2). Median EFS was not reached in the low-risk group, compared with 47.5 months (95% CI, 24.1–NR) in the intermediate-risk group and 18.0 months (95% CI, 12.8–NR) in the high-risk group. The corresponding 2- and 5-year EFS rates were 75% (95% CI, 66–85) and 56% (95% CI, 46–69) in the low-risk group, 60% (95% CI, 51–72) and 46% (95% CI, 37–59) in the intermediate-risk group, and 43% (95% CI, 31–60) and 33% (95% CI, 22–51) in the high-risk group, respectively (*p* = 0.004; Figure 3).

Internal validation using 1000 bootstrap resamples yielded an optimism-corrected c-index of 0.58. Time-dependent ROC analyses showed AUC values of 0.63, 0.64, 0.61, and 0.61 for prediction of 1-, 2-, 4-, and 5-year EFS, respectively, consistent with moderate discriminative performance. Calibration plots at 1, 2, 4, and 5 years showed acceptable agreement between predicted and observed EFS probabilities (Supplementary Figures S3 and S4).

## 4. Discussion

The lack of validated baseline tools to refine prognostic stratification in patients with MIBC treated with NAC remains an important clinical and translational challenge. Although cisplatin-based NAC followed by RC is a standard treatment for cisplatin-eligible patients, a substantial proportion of patients experience recurrence or early disease-related events despite multimodal therapy. In this context, easily accessible biomarkers capable of improving baseline risk assessment may help identify patients who require closer monitoring or evaluation of alternative perioperative strategies. In the present study, we developed an exploratory clinicopathological nomogram incorporating four baseline variables—sex, prior non–NMIBC, NLR, and age—to estimate 2- and 5-year EFS in patients with MIBC treated with cisplatin-based NAC.

The proposed model stratified patients into three clinically distinct risk groups. Low- and intermediate-risk patients showed more favorable outcomes after NAC, with median EFS not reached and 47.5 months, respectively, and pCR rates of approximately 31–43%. In contrast, high-risk patients had a median EFS of 18 months and a lower pCR rate of 20.9%, suggesting a subgroup with poorer outcomes despite standard NAC.

Systemic inflammation has emerged as a relevant biological and prognostic feature in bladder cancer. Among inflammatory biomarkers, pretreatment NLR is one of the most reproducible and clinically accessible parameters. A high NLR may reflect a tumor-promoting systemic inflammatory state, characterized by relative neutrophilia and/or lymphopenia, which could contribute to impaired antitumor immune surveillance, enhanced tumor progression, and reduced treatment sensitivity. Previous retrospective studies have shown that elevated NLR, commonly using cutoffs around 2–3, is associated with shorter survival in patients with urothelial carcinoma [23,24]. In addition, dynamic decreases in NLR during NAC have been associated with improved outcomes [25]. In line with these observations, our study identified baseline NLR as an independent adverse prognostic factor for EFS and supports its incorporation into pragmatic risk models for patients receiving cisplatin-based NAC.

To our knowledge, this is one of the first nomograms integrating a systemic inflammatory biomarker with baseline clinicopathological variables to estimate survival outcomes in NAC-treated MIBC. Previous models have mainly relied on clinical and pathological features, including tumor stage, histology, tumor size, and nodal status [14,15,26]. Compared with our previous bladder cancer-specific survival (BCSS) nomogram, the current model incorporates NLR and focuses specifically on EFS, an endpoint that captures recurrence, progression, inability to complete definitive surgery, and death. Conversely, histological variants and hydronephrosis, which were retained in our previous model [21], were not selected in the present analysis, possibly reflecting differences in cohort composition, stricter eligibility criteria, endpoint definition, and statistical selection procedures.

Several findings from the present study are consistent with previous evidence. Patients with prior NMIBC showed poorer outcomes after NAC, supporting the concept that secondary MIBC may represent a biologically distinct disease entity. Genomic studies have suggested that primary and secondary MIBC may differ in molecular alterations and evolutionary trajectories, which may partly explain differences in response to cisplatin-based chemotherapy [27]. Similarly, our observation that low pretreatment NLR was associated with better outcomes is consistent with prior studies linking systemic inflammatory status with survival and response to NAC [23,28].

The prognostic role of sex and age requires more cautious interpretation. Although female sex was retained in the final model, women represented only 11% of the cohort, limiting the precision and generalizability of this estimate. Previous studies evaluating the impact of sex on NAC response and survival have yielded mixed results, with some reporting differences in presentation stage, response, or outcomes, while others have not confirmed an independent effect [29,30]. Similarly, the association between age and response to NAC remains controversial. Some reports suggest comparable benefit from cisplatin-based NAC across age groups, whereas others indicate lower response rates or poorer outcomes in older patients [31,32]. In our model, age was retained during variable selection, but its effect size was modest and should not be interpreted as a causal determinant of outcome.

Interestingly, clinical TNM stage was not retained in the final model despite its established prognostic relevance in MIBC. Higher tumor stage and nodal involvement are known to be associated with worse outcomes after NAC and RC [1]. However, the distribution of advanced disease in our cohort may have limited the ability to detect an independent effect of clinical stage. In particular, only a minority of patients had T4 or clinically node-positive disease, and clinical staging before RC remains imperfect. These factors may have attenuated the prognostic contribution of cTNM in the multivariable model. This observation does not diminish the established prognostic relevance of disease burden. In this regard, recent studies have highlighted the value of additional nodal metrics, such as lymph node ratio, for refining prognostic stratification in node-positive patients treated with radical cystectomy and chemotherapy [33].

The present study should also be interpreted in the context of the changing perioperative treatment landscape for MIBC. The addition of immunotherapy to cisplatin-based NAC has recently demonstrated improved clinical outcomes, establishing chemo-immunotherapy as a new paradigm in the standard management of cisplatin-eligible patients [3]. In this setting, baseline inflammatory biomarkers such as NLR may become even more relevant, as they may capture host immune status and systemic inflammation, both of which could influence response to chemo-immunotherapy. However, the present nomogram was developed in a cohort treated before the routine incorporation of perioperative immunotherapy. Therefore, its applicability to contemporary cohorts receiving chemo-immunotherapy, antibody–drug conjugates, or other intensified perioperative approaches requires dedicated validation.

This study has several limitations. First, its retrospective design may introduce selection bias and residual confounding. Second, the sample size was modest, particularly for some subgroups such as women and patients with clinically node-positive disease. Third, model discrimination was moderate, with a Harrell’s c-index of 0.60 and an optimism-corrected c-index of 0.58, indicating that the nomogram provides only limited individual-level predictive accuracy. Fourth, potentially relevant variables, including smoking status, molecular subtype, genomic alterations, circulating tumor DNA, and additional inflammatory or immune-related biomarkers, were not available or were not formally evaluated. Although NLR was selected a priori based on its clinical accessibility, biological interpretability, and supporting evidence in the neoadjuvant setting, other inflammation-based indices, such as PLR, SII, and derived NLR, were not formally compared in the present study. Fifth, only internal validation was performed, and external validation in independent multicenter cohorts is required before clinical implementation. Finally, because all patients received NAC, the model cannot determine whether high-risk patients derive less benefit from NAC compared with upfront RC or alternative strategies.

### Future Directions

As the present nomogram was developed in a cohort of patients treated with NAC alone, its applicability to contemporary treatment strategies, including perioperative chemo-immunotherapy, remains uncertain. Although recent evidence in urothelial carcinoma treated with immune checkpoint inhibitors suggests that elevated pretreatment inflammatory biomarkers, particularly NLR, retain prognostic value in ICI-based settings, most available data derive from locally advanced or metastatic disease [34,35]. To our knowledge, evidence regarding the prognostic role of NLR in patients receiving neoadjuvant chemo-immunotherapy for MIBC remains limited, likely reflecting the recent incorporation of these regimens into clinical practice. Therefore, external validation in independent multicenter cohorts reflecting current clinical practice is required before clinical implementation.

Future studies should evaluate the model’s discrimination, calibration, and ability to stratify patients into clinically meaningful risk groups across different treatment settings. Prospective observational studies and analyses using trial-derived datasets, if available, may provide valuable opportunities to assess the reproducibility and clinical utility of the model in modern treatment contexts.

## 5. Conclusions

In conclusion, we developed and internally validated an exploratory clinicopathological nomogram integrating NLR, sex, age, and history of NMIBC to estimate EFS in patients with MIBC treated with cisplatin-based NAC followed by radical cystectomy. The model stratified patients into clinically distinct risk groups, with high-risk patients showing shorter EFS and lower pCR rates despite NAC.

Importantly, NLR is an inexpensive and routinely available inflammatory biomarker, supporting its potential value as part of a pragmatic baseline risk assessment. Nevertheless, the model showed only moderate discrimination and was internally validated only. Therefore, this nomogram should be regarded as hypothesis-generating and should not be used for individual treatment decisions before external validation.

Future studies should assess its performance in independent multicenter cohorts and contemporary treatment contexts, including perioperative chemo-immunotherapy and other intensified strategies.

## Figures and Tables

**Table 1:** Baseline characteristics, treatment-related variables, and pathological outcomes of the study cohort. dd-MVAC, dose-dense methotrexate, vinblastine, doxorubicin, and cisplatin; NAC, neoadjuvant chemotherapy; NLR, neutrophil-to-lymphocyte ratio; NMIBC, non–muscle-invasive bladder cancer; TNM, tumor-node-metastasis; TUR, transurethral resection.

Characteristic	Category	Overall Cohort, n (%)
**Demographic and clinical characteristics**		**N = 210**
**Sex**	Male	187 (89.0)
	Female	23 (11.0)
**Age, years**	<65	78 (37.1)
	65–75	100 (47.6)
	>75	32 (15.2)
**Histology**	Urothelial	176 (83.8)
	Variant histology	34 (16.2)
**Lymphovascular invasion**	Yes	47 (22.4)
	No	163 (77.6)
**Hydronephrosis**	Yes	63 (30.0)
	No	147 (70.0)
**Prior NMIBC**	Yes	28 (13.3)
	No	182 (86.7)
**Clinical TNM stage**	cT2N0M0	64 (30.5)
	cT3–4aN0M0	123 (58.6)
	cT2–4aN+M0	23 (11.0)
**Laboratory variables**		**N = 210**
**Baseline hemoglobin**	<12 g/dL	51 (24.3)
	≥12 g/dL	159 (75.7)
**Baseline platelet count**	<300 × 10^9^/L	140 (66.7)
	≥300 × 10^9^/L	70 (33.3)
**Neutrophil-to-lymphocyte ratio**	<2.78	104 (49.5)
	≥2.78	106 (50.5)
**Time from TUR to NAC initiation**	<6 weeks	107 (51.0)
	≥6 weeks	103 (49.0)
**Treatment-related variables**		**N = 210**
**NAC regimen**	Cisplatin/gemcitabine	195 (92.9)
	dd-MVAC	15 (7.1)
**NAC cycles**	1–3	185 (88.1)
	≥4	25 (11.9)
**Cystectomy status**	Complete resection	190 (90.5)
	Incomplete resection	13 (6.2)
	No surgery	7 (3.3)
**Pathological outcomes**		**N = 203**
**Pathological TNM stage**	ypT0N0M0	68 (33.5)
	ypT1N0M0	29 (14.3)
	ypT2–4N0M0	66 (32.5)
	ypT0–4N+M0	40 (19.7)

**Table 2:** Multivariable Cox regression analysis of baseline variables associated with event-free survival in patients with MIBC treated with NAC followed by radical cystectomy. Harrell’s c-index = 0.60 (95% CI, 0.54–0.66); optimism-corrected c-index, bootstrap mean = 0.584. CI, confidence interval; HR, hazard ratio; NLR, neutrophil-to-lymphocyte ratio; NMIBC, non–muscle-invasive bladder cancer; TNM, tumor-node-metastasis; TUR, transurethral resection.

Variable	Category	Multivariable Model HR (95% CI, *p* Value)	Final Prognostic Model HR (95% CI, *p* Value)
**Sex**	Male		
	Female	1.76 (0.99–3.11, *p* = 0.052)	1.76 (1.01–3.05, *p* = 0.045)
**Age, years**		0.98 (0.96–1.01, *p* = 0.159)	0.98 (0.96–1.01, *p* = 0.149)
**Clinical TNM stage**	cT2N0M0		
	cT3–4a/N0–1M0	1.12 (0.71–1.77, *p* = 0.612)	
**Hydronephrosis**	Yes		
	No	0.85 (0.54–1.34, *p* = 0.488)	
**Lymphovascular invasion**	Yes		
	No	0.90 (0.56–1.45, *p* = 0.665)	
**Prior NMIBC**	Yes		
	No	0.64 (0.38–1.10, *p* = 0.109)	0.62 (0.37–1.03, *p* = 0.067)
**Histology**	Urothelial		
	Variant histology	1.26 (0.74–2.15, *p* = 0.390)	
**Baseline hemoglobin**	<12 g/dL		
	≥12 g/dL	0.94 (0.57–1.54, *p* = 0.793)	
**NLR**	<2.78		
	≥2.78	1.59 (1.06–2.37, *p* = 0.023)	1.61 (1.09–2.37, *p* = 0.016)
**Baseline platelet count**	<300 × 10^9^/L		
	≥300 × 10^9^/L	0.88 (0.57–1.35, *p* = 0.555)	
**Time from TUR to NAC initiation**	<6 weeks		
	≥6 weeks	1.32 (0.88–1.97, *p* = 0.178)	

## Data Availability

Data are freely available upon reasonable request to the corresponding author.

## Supplementary Materials

The following supporting information can be downloaded at: https://www.mdpi.com/article/10.3390/cancers18132054/s1; Table S1: Baseline characteristics and treatment-related variables according to pathological response; Table S2: Median overall survival by baseline characteristics and treatment in patients with MIBC treated with NAC followed by RC; Table S3: Median event-free survival by baseline characteristics and treatment in patients with MIBC treated with NAC followed by RC; Figure S1. Assessment of the proportional hazards assumption using scaled Schoenfeld residuals. Scaled Schoenfeld residual plots are shown for the four variables included in the final Cox model: sex, age, prior non–muscle-invasive bladder cancer (NMIBC), and neutrophil-to-lymphocyte ratio (NLR). No significant violation of the proportional hazards assumption was observed for any individual variable or for the global test (*p* = 0.119). Figure S2: Kaplan–Meier estimates of overall survival (A) and event-free survival (B) according to pathological response in patients with muscle-invasive bladder cancer treated with neoadjuvant chemotherapy followed by radical cystectomy; Figure S3: Calibration plots of the nomogram for event-free survival at 1 year (A), 2 years (B), 4 years (C), and 5 years (D) in patients with muscle-invasive bladder cancer treated with neoadjuvant chemotherapy followed by radical cystectomy; Figure S4: Time-dependent receiver operating characteristic curves for the nomogram predicting event-free survival at 1 year (A), 2 years (B), 4 years (C), and 5 years (D) in patients with muscle-invasive bladder cancer treated with neoadjuvant chemotherapy followed by radical cystectomy.

## Abbreviations

The following abbreviations are used in this manuscript:

AIC	Akaike information criterion
AJCC	American Joint Committee on Cancer
AUC	area under the curve
BCSS	bladder cancer-specific survival
CI	confidence interval
cTNM	clinical tumor–node–metastasis stage
CT	computed tomography
dd-MVAC	dose-dense methotrexate, vinblastine, doxorubicin, and cisplatin
EFS	event-free survival
HR	hazard ratio
IQR	interquartile range
LVI	lymphovascular invasion
MIBC	muscle-invasive bladder cancer
NAC	neoadjuvant chemotherapy
NLR	neutrophil-to-lymphocyte ratio
NMIBC	non–muscle-invasive bladder cancer
NR	not reached
OS	overall survival
pCR	pathological complete response
pTNM	pathological tumor–node–metastasis stage
RC	radical cystectomy
ROC	receiver operating characteristic
TUR	transurethral resection of bladder tumor
TTNAC	time from transurethral resection to neoadjuvant chemotherapy initiation
ypTNM	post-neoadjuvant pathological tumor–node–metastasis stage
