# Supplementary material for: Integrating the Neutrophil-to-Lymphocyte Ratio into a Clinicopathological Nomogram for Event-Free Survival Prediction in Cisplatin-Treated Muscle-Invasive Bladder Cancer
Source: Cancers (Basel). 2026 Jun 24;18(13):2054. doi: 10.3390/cancers18132054 (PMC13359792; doi:10.3390/cancers18132054)
Supplement: Supplementary file 1 [file cancers-18-02054-s001.zip › cancers-4372826-supplementary.pdf]

Supplementary Table S1. Baseline characteristics and treatment-related variables according to pathological response.

Characteristic	Category	All (N = 203), n (%)	Pathological response Yes (N = 97), n (%)	Pathological response No (N = 106), n (%)	p value
**Demographic and clinical characteristics**					
**Sex**	Male	180 (88.7)	89 (91.8)	91 (85.8)	0.270
	Female	23 (11.3)	8 (8.2)	15 (14.2)	
**Age, years**	<65	73 (36.0)	33 (34.0)	40 (37.7)	0.820
	65–75	98 (48.3)	49 (50.5)	49 (46.2)	
	>75	32 (15.8)	15 (15.5)	17 (16.0)	
**Histology**	Urothelial	171 (84.2)	79 (81.4)	92 (86.8)	0.394
	Variant histology	32 (15.8)	18 (18.6)	14 (13.2)	
**Lymphovascular invasion**	Yes	46 (22.7)	21 (21.6)	25 (23.6)	0.872
	No	157 (77.3)	76 (78.4)	81 (76.4)	
**Hydronephrosis**	Yes	61 (30.0)	26 (26.8)	35 (33.0)	0.417
	No	142 (70.0)	71 (73.2)	71 (67.0)	
**Prior NMIBC**	Yes	28 (13.8)	11 (11.3)	17 (16.0)	0.444
	No	175 (86.2)	86 (88.7)	89 (84.0)	
**Clinical TNM stage**	cT2N0M0	62 (30.5)	33 (34.0)	29 (27.4)	0.385
	cT3–4aN0M0	119 (58.6)	56 (57.7)	63 (59.4)	
	cT2–4aN+M0	22 (10.8)	8 (8.2)	14 (13.2)	
**Laboratory variables**					
**Baseline hemoglobin**	<12 g/dL	49 (24.1)	22 (22.7)	27 (25.5)	0.764
	≥12 g/dL	154 (75.9)	75 (77.3)	79 (74.5)	
**Baseline platelet count**	<300 × 10^9/L	136 (67.0)	66 (68.0)	70 (66.0)	0.878
	≥300 × 10^9/L	67 (33.0)	31 (32.0)	36 (34.0)	
**Neutrophil-to-lymphocyte ratio**	<2.78	103 (50.7)	56 (57.7)	47 (44.3)	0.077
	≥2.78	100 (49.3)	41 (42.3)	59 (55.7)	
**Time from TUR to NAC initiation**	<6 weeks	104 (51.2)	49 (50.5)	55 (51.9)	0.956
	≥6 weeks	99 (48.8)	48 (49.5)	51 (48.1)	
**Treatment-related variables**					
**NAC regimen**	Cisplatin/gemcitabine	188 (92.6)	90 (92.8)	98 (92.5)	1.000
	dd-MVAC	15 (7.4)	7 (7.2)	8 (7.5)	
**NAC cycles**	1–3	179 (88.2)	83 (85.6)	96 (90.6)	0.377
	≥4	24 (11.8)	14 (14.4)	10 (9.4)	

Characteristic	Category	All (N = 203), n (%)	Pathological response Yes (N = 97), n (%)	Pathological response No (N = 106), n (%)	p value
**Cystectomy status**	Complete resection	190 (93.6)	96 (99.0)	94 (88.7)	0.007
	Incomplete resection	13 (6.4)	1 (1.0)	12 (11.3)	

dd-MVAC, dose-dense methotrexate, vinblastine, doxorubicin, and cisplatin; MIBC, muscle-invasive bladder cancer; NAC, neoadjuvant chemotherapy; NLR, neutrophil-to-lymphocyte ratio; NMIBC, non–muscle-invasive bladder cancer; RC, radical cystectomy; TUR, transurethral resection; TTNAC, time from TUR to NAC initiation.

Supplementary Table S2. Median overall survival by baseline characteristics and treatment in patients with MIBC treated with NAC followed by RC.

Characteristic	Category	Median OS (95% CI)	p value
**All patients**		58.8 (47.5-NR)	
**Demographic and clinical characteristics**			
**Sex**	Male	NR (49.5-NR)	0.11
	Female	26.5 (16.0-NR)	
**Age, years**	<65	57.8 (26.5-NR)	0.68
	65-75	58.8 (45.4-NR)	
	>75	NR (40.3-NR)	
**Histology**	Urothelial	58.8 (47.5-NR)	0.61
	Variant histology	NR (26.4-NR)	
**Lymphovascular invasion**	Yes	47.5 (32.8-NR)	0.90
	No	58.8 (48.5-NR)	
**Hydronephrosis**	Yes	47.5 (25.3-NR)	0.29
	No	NR (48.8-NR)	
**Prior NMIBC**	Yes	33.6 (22.2-NR)	0.11
	No	NR (48.8-NR)	
**Laboratory variables**			
**Baseline hemoglobin**	<12 g/dL	45.9 (23.1-NR)	0.22
	>=12 g/dL	NR (48.8-NR)	
**Baseline platelet count**	<300 x 10^9/L	51.8 (40.4-NR)	0.35
	>=300 x 10^9/L	NR (47.5-NR)	
**Neutrophil-to-lymphocyte ratio**	<2.78	NR (54.8-NR)	0.04
	>=2.78	40.9 (26.5-NR)	
**Time from TUR to NAC initiation**	<6 weeks	NR (47.5-NR)	0.24
	>=6 weeks	51.8 (34.3-NR)	
**Treatment-related variables**			
**Clinical TNM stage**	cT2N0M0	NR (40.9-NR)	0.47
	cT3-4aN0M0	57.5 (37.3-NR)	
	cT2-4aN+M0	NR (40.4-NR)	
**NAC regimen**	Cisplatin/gemcitabine	NR (47.5-NR)	0.85
	dd-MVAC	55.6 (22.0-NR)	
**NAC cycles**	1-3	57.8 (40.9-NR)	0.19
	>=4	NR (44.1-NR)	
**Cystectomy status**	Complete resection	NR (55.6-NR)	<0.0001

Characteristic	Category	Median OS (95% CI)	p value
	Incomplete resection	14.7 (9.2-NR)	
	No surgery	9.2 (2.5-NR)	
**Pathological outcomes**			
**Pathological response**	Yes	NR (NR-NR)	<0.0001

CI, confidence interval; dd-MVAC, dose-dense methotrexate, vinblastine, doxorubicin, and cisplatin; MIBC, muscle-invasive bladder cancer; NAC, neoadjuvant chemotherapy; NLR, neutrophil-to-lymphocyte ratio; NMIBC, non-muscle-invasive bladder cancer; NR, not reached; OS, overall survival; RC, radical cystectomy; TUR, transurethral resection; TTNAC, time from TUR to NAC initiation.

Supplementary Table S3. Median event-free survival by baseline characteristics and treatment in patients with MIBC treated with NAC followed by RC.

Characteristic	Category	Median EFS (95% CI)	p value
**All patients**		48.5 (37.7-NR)	
**Demographic and clinical characteristics**			
**Sex**	Male	55.7 (40.9-NR)	0.04
	Female	15.6 (11.4-NR)	
**Age, years**	<65	45.9 (22.8-NR)	0.63
	65-75	52.9 (30.2-NR)	
	>75	NR (19.9-NR)	
**Histology**	Urothelial	54.6 (40.3-NR)	0.22
	Variant histology	29.8 (12.8-NR)	
**Lymphovascular invasion**	Yes	45.9 (17.2-NR)	0.45
	No	54.6 (38.8-NR)	
**Hydronephrosis**	Yes	45.7 (20.8-NR)	0.51
	No	54.6 (38.8-NR)	
**Prior NMIBC**	Yes	23.5 (15.9-NR)	0.16
	No	55.7 (40.9-NR)	
**Laboratory variables**			
**Baseline hemoglobin**	<12 g/dL	45.9 (18.9-NR)	0.30
	>=12 g/dL	55.7 (37.7-NR)	
**Baseline platelet count**	<300 x 10^9/L	45.9 (30.2-NR)	0.66
	>=300 x 10^9/L	57.8 (28.4-NR)	
**Neutrophil-to-lymphocyte ratio**	<2.78	NR (45.9-NR)	0.03
	>=2.78	32.0 (19.0-NR)	
**Time from TUR to NAC initiation**	<6 weeks	NR (40.3-NR)	0.29
	>=6 weeks	44.7 (26.9-NR)	
**Treatment-related variables**			
**Clinical TNM stage**	cT2N0M0	55.7 (37.7-NR)	0.53
	cT3-4aN0M0	45.7 (26.9-NR)	
	cT2-4aN+M0	NR (19.0-NR)	
**NAC regimen**	Cisplatin/gemcitabine	48.5 (36.9-NR)	0.98
	dd-MVAC	52.9 (17.3-NR)	
**NAC cycles**	1-3	47.5 (30.2-NR)	0.18
	>=4	NR (39.2-NR)	

Characteristic	Category	Median EFS (95% CI)	p value
**Cystectomy status**	Complete resection	NR (45.7-NR)	<0.0001
	Incomplete resection	10.8 (7.9-NR)	
	No surgery	4.7 (2.5-NR)	
**Pathological outcomes**			
**Pathological response**	Yes	NR (NR-NR)	<0.0001
	No	17.0 (15.4-28.4)	

CI, confidence interval; dd-MVAC, dose-dense methotrexate, vinblastine, doxorubicin, and cisplatin; EFS, event-free survival; MIBC, muscle-invasive bladder cancer; NAC, neoadjuvant chemotherapy; NLR, neutrophil-to-lymphocyte ratio; NMIBC, non-muscle-invasive bladder cancer; NR, not reached; RC, radical cystectomy; TUR, transurethral resection; TTNAC, time from TUR to NAC initiation.

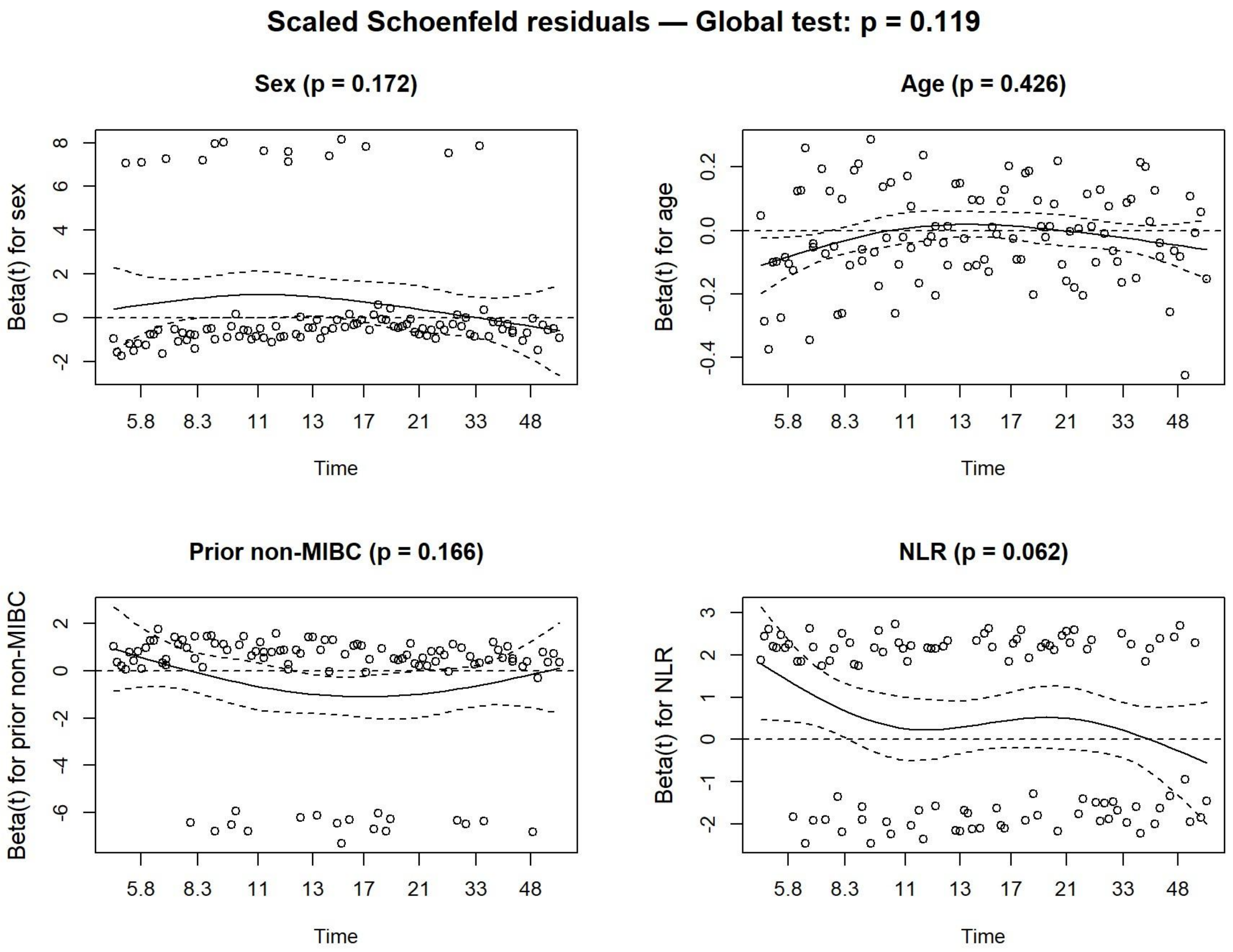

Figure S1. Assessment of the proportional hazards assumption using scaled Schoenfeld residuals. Scaled Schoenfeld residual plots are shown for the four variables included in the final Cox model: sex, age, prior non–muscle-invasive bladder cancer (NMIBC), and neutrophil-to-lymphocyte ratio (NLR). No significant violation of the proportional hazards assumption was observed for any individual variable or for the global test (p = 0.119).

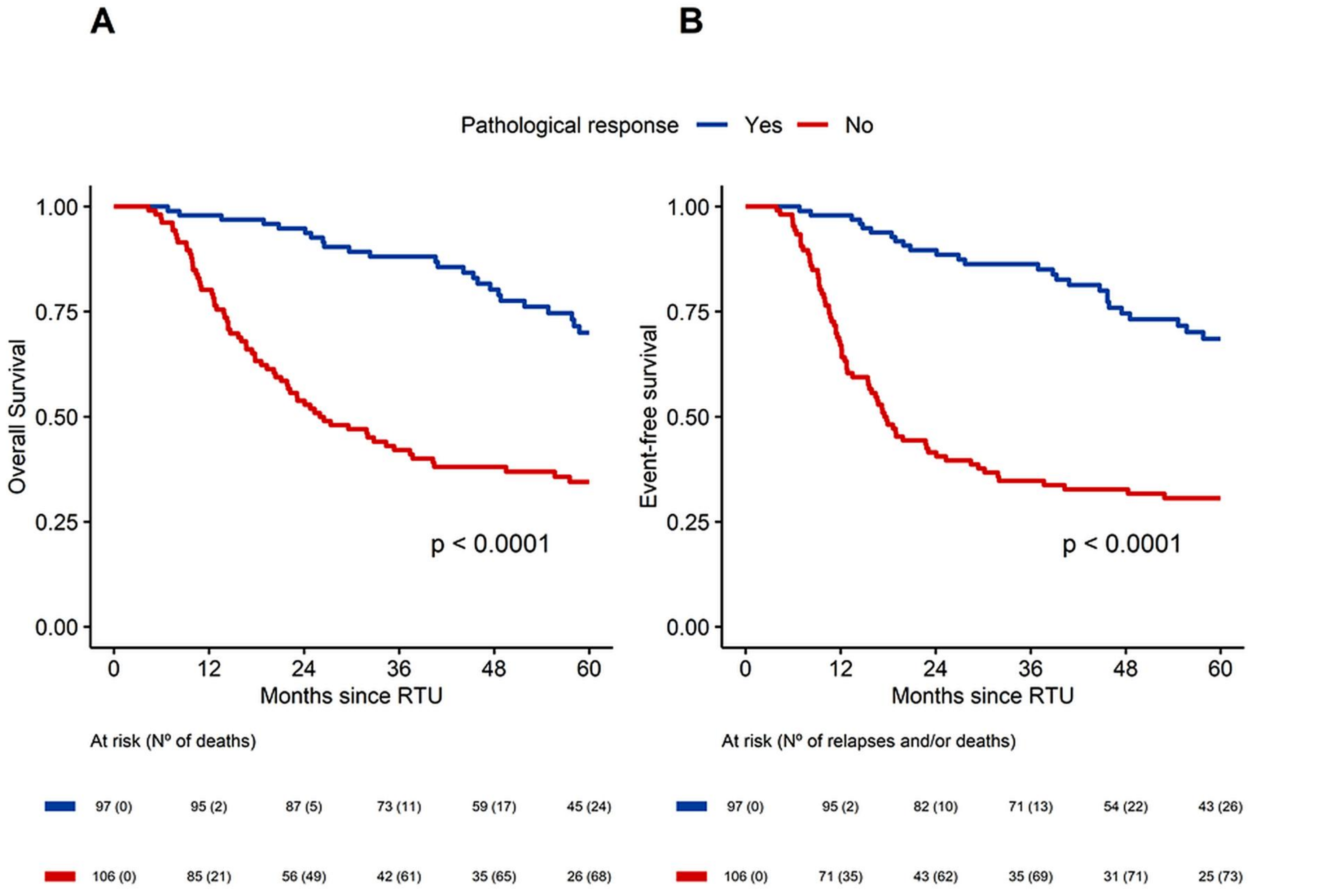

Figure S2. Kaplan–Meier estimates of overall survival (A) and event-free survival (B) according to pathological response in patients with muscle-invasive bladder cancer treated with neoadjuvant chemotherapy followed by radical cystectomy.

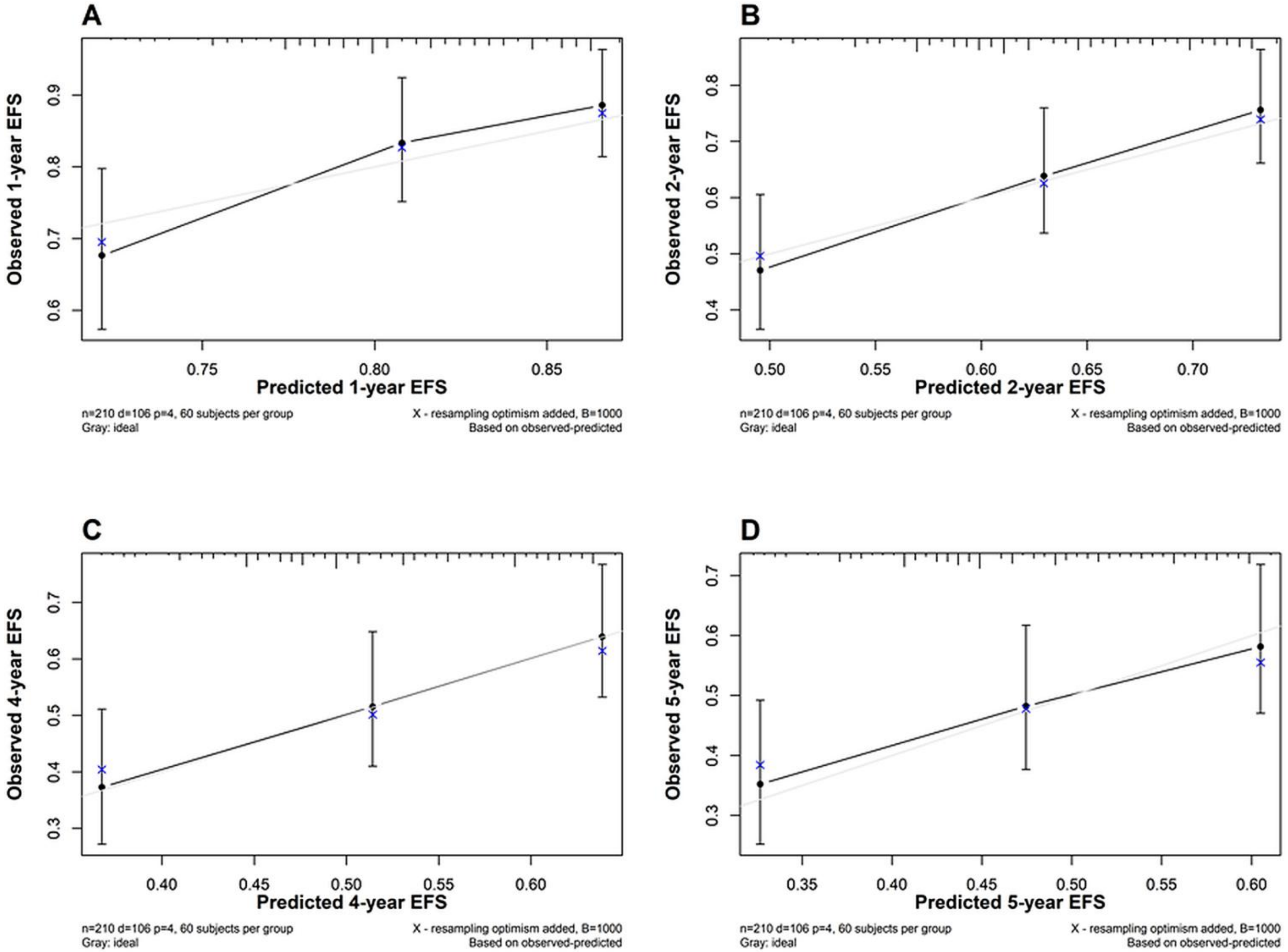
A
Observed 1-year EFS
Predicted 1-year EFS
0.75
0.80
0.85
0.6
0.7
0.8
0.9
n=210 d=106 p=4, 60 subjects per group
Gray: ideal
X - resampling optimism added, B=1000
Based on observed-predicted
B
Observed 2-year EFS
Predicted 2-year EFS
0.50
0.55
0.60
0.65
0.70
0.4
0.5
0.6
0.7
0.8
n=210 d=106 p=4, 60 subjects per group
Gray: ideal
X - resampling optimism added, B=1000
Based on observed-predicted
C
Observed 4-year EFS
Predicted 4-year EFS
0.40
0.45
0.50
0.55
0.60
0.3
0.4
0.5
0.6
0.7
n=210 d=106 p=4, 60 subjects per group
Gray: ideal
X - resampling optimism added, B=1000
Based on observed-predicted
D
Observed 5-year EFS
Predicted 5-year EFS
0.35
0.40
0.45
0.50
0.55
0.60
0.3
0.4
0.5
0.6
0.7
n=210 d=106 p=4, 60 subjects per group
Gray: ideal
X - resampling optimism added, B=1000
Based on observed-predicted

Figure S3. Calibration plots of the nomogram for event-free survival at 1 year (A), 2 years (B), 4 years (C), and 5 years (D) in patients with muscle-invasive bladder cancer treated with neoadjuvant chemotherapy followed by radical cystectomy.

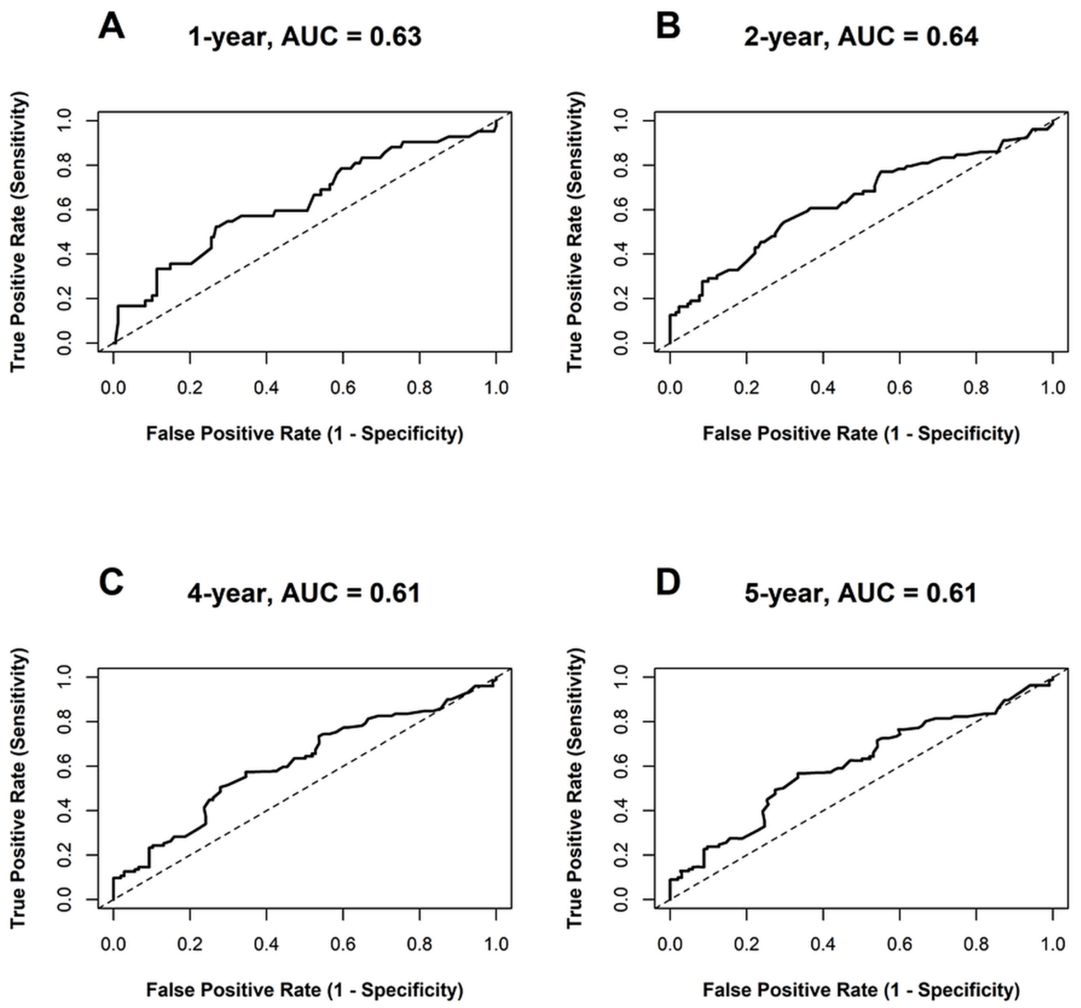

Figure S4. Time-dependent receiver operating characteristic curves for the nomogram predicting event-free survival at 1 year (A), 2 years (B), 4 years (C), and 5 years (D) in patients with muscle-invasive bladder cancer treated with neoadjuvant chemotherapy followed by radical cystectomy.
